# Enhanced thrombin generation potential and endothelial dysfunction in chronic spontaneous urticaria

**DOI:** 10.1002/clt2.12285

**Published:** 2023-07-21

**Authors:** Katie Ridge, Helen Fogarty, Dearbhla Doherty, Mary Byrne, Cliona O’Farrelly, James S. O’Donnell, Niall Conlon

**Affiliations:** ^1^ UCARE Centre Department of Immunology St. James's Hospital Dublin Ireland; ^2^ School of Medicine Trinity College Dublin Dublin Ireland; ^3^ Irish Centre of Vascular Biology School of Pharmacy and Biomolecular Sciences Royal College of Surgeons in Ireland Dublin Ireland; ^4^ National Coagulation Centre St. James's Hospital Dublin Ireland; ^5^ Comparative Immunology Group School of Immunology and Biochemistry Trinity College Dublin Dublin Ireland

To the Editor,

Chronic spontaneous urticaria (CSU) is characterized by recurrent hives that last longer than 6 weeks. The relationship between the coagulation cascade, endothelial cell (EC) activation and urticaria pathogenesis is acknowledged but remains poorly understood.^1^, ^2^ Examination of these pathways may offer opportunities for improved disease endotyping, prognostication and novel therapeutic avenues.

Mast cells and eosinophils are known to be important in CSU pathogenesis.^3^, ^4^ Mast cell degranulation results in the generation of leukotrienes and mast cell‐derived mediators. Resulting EC activation promotes vascular permeability. Activated eosinophils express tissue factor, which initiates coagulation via Factor VII.^3^ Despite our understanding of these pathways, the distinct profiles of coagulation and fibrinolysis in CSU remain obscure. Furthermore, products of fibrinolysis, such as D‐Dimer, are increasingly proposed as potential markers of severe disease.^4^ This study sought to examine markers of EC activation as well as the dynamics of thrombin generation (TG) in patients with CSU.

Adult patients with a diagnosis of CSU attending an urticaria clinic at a tertiary Irish teaching Hospital were invited to participate. The study received ethical approval and informed written consent was obtained. Patients (*N* = 26) with CSU completed the urticaria control test, a measure of disease control assessing hives and swellings over the past 4 weeks.^5^ In this four item questionnaire with scores of 0–16, lower scores indicate higher symptom burden. A healthy control group without a diagnosis of CSU was recruited (*N* = 18). Markers of EC activation included plasma von Willebrand Factor (VWF) antigen (VWF:Ag) and procoagulant Factor VIII (FVIII:C) levels. These assays, in conjunction with a thrombin generation assay (TGA), were performed on participant plasma as previously described.^6^ Statistical analyses were performed using *t* tests in GraphPad Prism 9.0 (GraphPad Software) with a *p* value of < 0.05 considered statistically significant.

We assessed levels of TG, plasma FVIII:C and plasma VWF:Ag and compared them with levels found in the control group. We also compared patients with CSU who were in receipt of anti‐IgE therapy and high dose antihistamines with CSU patients on high dose antihistamines only.

Baseline characteristics are outlined in Table 1. CSU patients had a distinct TG profile relative to controls with an increased peak thrombin (*p* ≤ 0.0001) shortened time to peak thrombin (*p* = 0.004) and an enhanced velocity index (*p* = 0.0001) (see Figure 1). There was a trend towards elevated endogenous thrombin potential (ETP) in patients with CSU although this was not statistically significant (*p* = 0.07). Neither age and gender nor the presence of angioedema significantly influenced TG parameters.

**TABLE 1:** Baseline characteristics of CSU patients and controls.

	CSU (*n* = 26)	Controls (*n* = 18)
Gender	19F:7M	12F:6M
Age (*x̄*, SD)	42.9 years (12.3)	38.6 years (7.9)
Disease duration (*x̄*, SD)	6.8 years (6.85)	
Angioedema (*n*, %)	17 (65%)	
Peripheral basopenia^a^ (*n*, %)	12 (46%)	
UCT score (*x̄*, SD)	7.2 (5.8)	
On anti IgE treatment (*n*, %)	7/26 (27%)	
Median FVIII:C (IU/mL)	1.12	
Median VWF:Ag (IU/mL)	1.07	

Abbreviations: CSU, chronic spontaneous urticaria.

^a^ Peripheral basopenia defined as 0.0 basophils per 10^9^ per liter.

**FIGURE 1:**
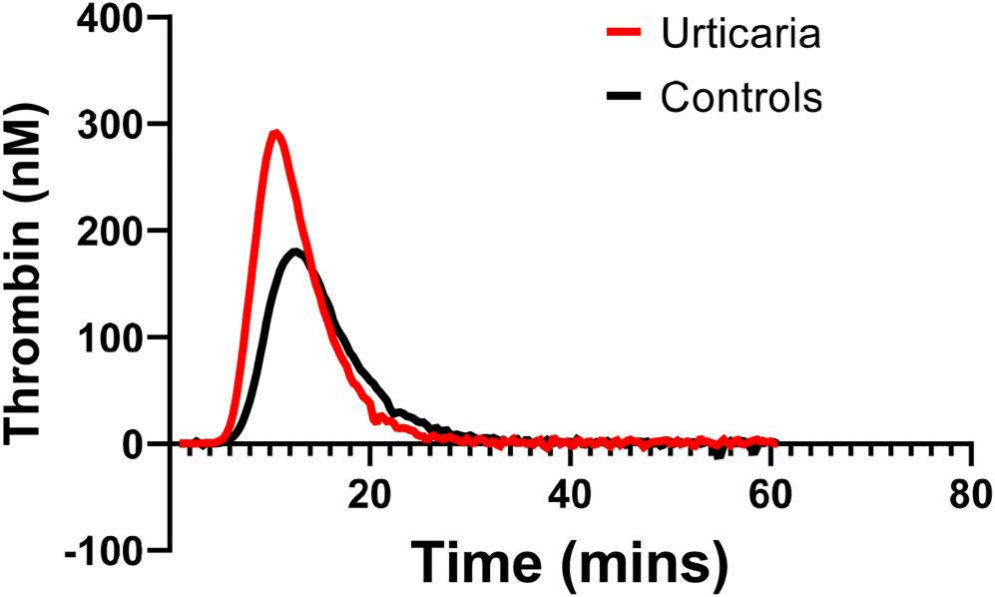
Combined thrombin generation curve for patients with chronic spontaneous urticaria (*N* = 26) and healthy controls (*N* = 18).

Elevated plasma FVIII:C levels are known to influence TG. We assessed plasma FVIII:C (*n* = 20). The reference range for FVIII:C is 0.60–1.36 IU/mL. Six of 20 patients with CSU had a FVIII:C level above 1.36 IU/mL. FVIII:C levels correlated with ETP (*p* < 0.0001), peak thrombin (*p* < 0.0001), time to peak thrombin (*p* < 0.0001) and lag time (*p* < 0.0001), suggesting that increased plasma FVIII:C levels may contribute towards increased TG in patients with CSU.

To further investigate the elevated FVIII:C seen in some CSU samples, we assessed plasma VWF:Ag (*n* = 21). The majority of FVIII:C circulates in normal plasma complexed with VWF. Both factors are predominantly synthesized by ECs, and VWF is a marker of acute and chronic endothelial activation.^7^ VWF:Ag levels correlated strongly with FVIII:C levels (*r* = 0.759, *p* < 0.001) and seven out of 20 patients with CSU had a VWF:Ag levels above the upper limit of normal (0.50–1.50 IU/mL).

We compared TG in patients with CSU who were receiving anti‐IgE therapy with those who were not. We found that patients who were not receiving anti‐IgE therapy had significantly shorter lag times than those who were receiving anti‐ IgE therapy (*p* = 0.0264, 95% confidence interval −2.903 to −0.2045). This implies that patients who were on anti‐IgE therapy took longer to generate thrombin. A previous study on the effect of anti‐IgE therapy on TG in CSU found a significant effect on Factor 1 and Factor 2 with no other significant change in TGA parameters.^8^ Future research may benefit from examining the links between disease control and TG profiles.

Despite the interpretive constraints of our small sample size, this study identifies for the first time, a relationship between VWF:Ag, FVIII:C and TG in CSU. Elevated plasma VWF has been reported as a marker of EC dysfunction in a range of clinical conditions reflecting acute and chronic endothelial activation.^6^, ^7^, ^9^ Our findings point to significantly enhanced TG potential and endothelial dysfunction in CSU. Importantly, VWF and FVIII:C levels correlated strongly with markers of TG, suggesting that EC activation may modulate TG in these patients. Patients with CSU do not have a reported increased clotting risk. Nevertheless, our data demonstrate significantly higher peak thrombin levels in patients with CSU compared to controls. Furthermore, patients who were receiving anti‐IgE therapy at the time of sampling had longer lag times, indicating that anti‐IgE therapy affects thrombin lagtime.

Plasma FVIII:C may be an important part of the inflammatory signal in CSU. Further interrogation of this parameter in CSU may improve our understanding of crosstalk between immune cell activation and the coagulation cascade. We propose that the TGA is a key tool for probing the complexities of coagulation and fibrinolysis in this cohort of patients. Further studies on biomarkers of endothelial dysfunction may help identify disease subgroups that could benefit from the manipulation of the coagulation system.

## AUTHOR CONTRIBUTIONS

Niall Conlon, Cliona O’Farrelly and James S. O’Donnell conceived the research plan. Helen Fogarty, Dearbhla Doherty and Mary Byrne performed laboratory experiments. Katie Ridge performed statistical analysis and wrote the manuscript. All authors reviewed and critiqued the final manuscript.

## CONFLICT OF INTEREST STATEMENT

The authors whose names are listed immediately below certify that they have NO affiliations with or involvement in any organization or entity with any financial interest (such as honoraria; educational grants; participation in speakers' bureaus; membership, employment, consultancies, stock ownership, or other equity interest; and expert testimony or patent‐licensing arrangements), or non‐financial interest (such as personal or professional relationships, affiliations, knowledge or beliefs) in the subject matter or materials discussed in this manuscript.

## FUNDING INFORMATION

Wellcome Trust and the Health Research Board, Grant/Award Number: 203930/B/16/Z; Health Service Executive, National Doctors Training and Planning; Health and Social Care, Research and Development Division, Northern Ireland.

## Data Availability

The data that support the findings of this study are available on request from the corresponding author. The data are not publicly available due to privacy or ethical restrictions.
